# Increased frequency and quantity of mucosal and plasma cytomegalovirus replication among Ugandan Adults Living with HIV

**DOI:** 10.1371/journal.pone.0287516

**Published:** 2023-08-04

**Authors:** Elisabeth McClymont, Jeffrey Bone, Jackson Orem, Fred Okuku, Mary Kalinaki, Misty Saracino, Meei-Li Huang, Stacy Selke, Anna Wald, Lawrence Corey, Corey Casper, Isabelle Boucoiran, Christine Johnston, Soren Gantt

**Affiliations:** 1 Department of Pediatrics, University of British Columbia, Vancouver, Canada; 2 CIHR Canadian HIV Trials Network, Vancouver, Canada; 3 Department of Obstetrics and Gynecology, University of British Columbia, Vancouver, Canada; 4 British Columbia Children’s Hospital Research Institute, Vancouver, Canada; 5 Uganda Cancer Institute, Mulago Hospital, Makerere University, Kampala, Uganda; 6 Department of Laboratory Medicine, University of Washington, Seattle, Washington, United States of America; 7 Vaccine and Infectious Disease Division, Fred Hutchinson Cancer Research Center, Seattle, Washington, United States of America; 8 Department of Medicine, University of Washington, Seattle, Washington, United States of America; 9 Department of Epidemiology, University of Washington, Seattle, Washington, United States of America; 10 Département d’Obstétrique-Gynécologie, Université de Montréal, Montréal, Canada; 11 Département de Microbiologie, Infectiologie et Immunologie, Université de Montréal, Montréal, Canada; University of Zambia School of Medicine, ZAMBIA

## Abstract

**Background:**

Co-infection with HIV can result in impaired control of cytomegalovirus (CMV) replication, increasing the likelihood of disease and onward transmission. The objective of this analysis was to measure the impact of HIV on CMV replication in an intensively-sampled cohort in Kampala, Uganda.

**Methods:**

CMV seropositive men and women aged 18–65, with or without HIV co-infection, were followed for one month. Daily oral swabs and weekly anogenital swabs and plasma were collected. Quantitative CMV PCR was performed on all samples.

**Results:**

Eighty-five participants were enrolled and provided ≥1 oral swab; 43 (51%) were HIV-seropositive. People living with HIV (PLWH; median CD4 count 439 cells/mm^3^; none on antiretrovirals) had 2–4 times greater risk of CMV detection at each anatomical site assessed. At the oral site, 773 of 1272 (61%) of samples from PLWH had CMV detected, compared to 214 of 1349 (16%) among people without HIV. Similarly, the mean CMV quantity was higher among PLWH at all anatomical sites, with the largest difference seen for oral swabs (mean difference 1.63 log/mL; 95% CI 1.13–2.13). Among PLWH, absolute quantity of CD4+ T-cells was not associated with risk of CMV detection. HIV plasma RNA quantity was positively correlated with oral CMV shedding frequency, but not detection at other sites.

**Conclusions:**

Mucosal and systemic CMV replication occurs at higher levels in PLWH than people without HIV, particularly oral shedding, which is a major mode of CMV transmission. Increased CMV replication despite relatively preserved CD4+ T-cell counts suggests that additional interventions are required to improve CMV control in PLWH.

## Background

Cytomegalovirus (CMV) infection is ubiquitous, particularly in low- and middle-income countries, where it is acquired nearly universally in early life [1–3]. CMV infection is particularly important in people living with HIV (PLWH) who are at high risk of complications, including sight- and life-threatening disease. Most CMV end-organ disease occurs in PLWH with an absolute quantity of CD4+ T-cells (CD4+ count) <50 cells/mm^3^, in whom CMV viremia is a well-described risk factor [4–6]. Even in the absence of overt disease, CMV viremia is associated with mortality in PLWH, independent of CD4+ count and HIV plasma RNA quantity (HIV viral load) [7–9]. CMV infection has also been associated with an increased risk of cardiovascular disease and all-cause mortality [10–12]. Finally, among PLWH, CMV co-infection causes immune dysregulation that may result in inferior responses to vaccines [13].

Data regarding CMV replication patterns in PLWH are relatively sparse, with most published studies using cross-sectional or limited sampling, often amid advanced HIV disease [14–18]. One study describing longitudinal vaginal CMV detection in women living with HIV documented CMV DNA in 78% of participants but did not assess other bodily sites [19].

### Objectives

Our objective was to comprehensively characterize the effect of HIV co-infection on mucosal and systemic CMV replication, leveraging intensively-collected samples from a cohort of Ugandan adults.

### Study design

Adults aged 18–65 with or without HIV were recruited for prospective studies of herpesvirus infections and Kaposi Sarcoma (KS) at the Uganda Cancer Institute in Kampala between May 2005 and July 2006 [20]. Eligible adults were not taking medications with anti-herpesvirus activity, and PLWH had a CD4+ count >200 cells/mm^3^ and were not taking ART, per WHO guidelines at the time [21]. Study procedures were approved by the Makerere University Research and Ethics Committee, the Uganda National Council for Science and Technology, and the University of Washington Human Subjects Division. Informed consent was obtained from all participants. Written consent was obtained when able, and witnessed verbal consent was obtained in place of written consent if participants were unable to read and understand the consent form.

Over 28 days, participants self-collected oral swabs daily [20]. Focused physical exams and collection of oral and anogenital swabs and plasma samples were performed by clinicians weekly. Commercial immunoassays were used for HIV (Inverness Medical Innovations, Inc) and CMV (Abbott Laboratories) serostatus. CD4+ count and HIV viral load were measured using standard cell sorting techniques and the Amplicor HIV-1 monitor test (Roche, version 1.5), respectively. DNA was extracted from mucosal swabs and plasma [22]. Real-time qPCR was performed using specific primers to detect gB and IE1 genes of CMV [23] with positive/negative controls [22,24]. Mucosal samples with >150 copies/ml and plasma samples with >50 copies/ml were considered positive [25].

Average rates of CMV mucosal shedding and viremia were calculated by anatomic site. Frequency of mucosal shedding and viremia were compared between people with and without HIV using generalized estimating equations (GEE) models with binomial log-links and exchangeable correlation structures to account for within-participant correlations. A Poisson log-link was substituted where log-binomial models failed to converge [26]. Analyses were run unadjusted and adjusted for KS status. Mean difference in shedding quantity was examined between those with and without HIV using a Gaussian GEE model. Among PLWH, we estimated the association of CD4+ T-cell count and HIV viral load with average CMV shedding rate for each site using similar GEE models. All analyses were performed using R v3.6.3 [27].

## Results

Eighty-five participants provided ≥1 oral swab. All participants had serologic or virologic evidence of CMV infection. Forty-three (51%) participants were HIV-seropositive (Table 1). Thirty-two (38%) participants were female. Median age was 32 years (range 18–60). Among PLWH, the median CD4+ count was 439 cells/mm^3^ (IQR: 324–596 cells/mm^3^) and the median HIV viral load was 55,727 copies/ml (IQR: 12,623–152,672 copies/ml). Participants collected 100% of expected oral and plasma samples, and 95% of expected genital swabs.

**Table 1:** Participant demographics and clinical characteristics by HIV co-infection status.

	HIV seronegative (N = 42)	HIV seropositive (N = 43)
Female	18 (43%)	14 (33%)
Age	31.0 (26.0–37.0)	32.0 (27.0–40.5)
KS positive	20 (48%)	21 (50%)
CD4 count, cells/mm^3^	-	439 (324–596)
HIV plasma RNA level, copies/ml	-	55,727 (12,623–152,672)
≥1 oral swab	42 (100%)	43 (100%)
Number of swabs per participant	33.0 [32.2, 34.0]	33.0 [26.0, 34.0]
≥1 genital swab	38 (91%)	41 (95%)
Number of swabs per participant	4 [4, 5]	4 [4, 5]
≥1 plasma sample	42 (100%)	42 (98%)
Number of samples per participant	5 [4, 6]	4 [4, 5]

Categorical data are N (%) and continuous are median [IQR].

**Fig 1:**
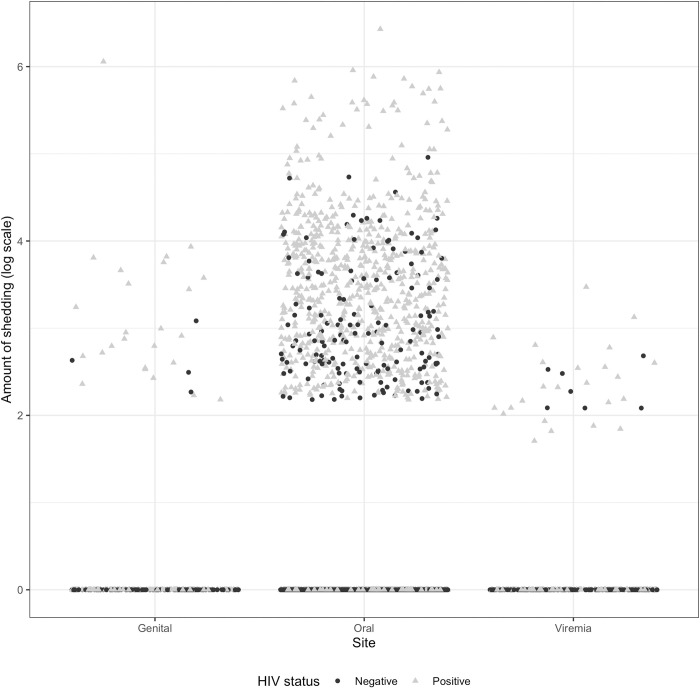
Quantity of CMV detected for all samples, by HIV status and anatomic site. The CMV qPCR results are shown for people without HIV in black circles and people living with HIV in grey triangles.

**Fig 2:**
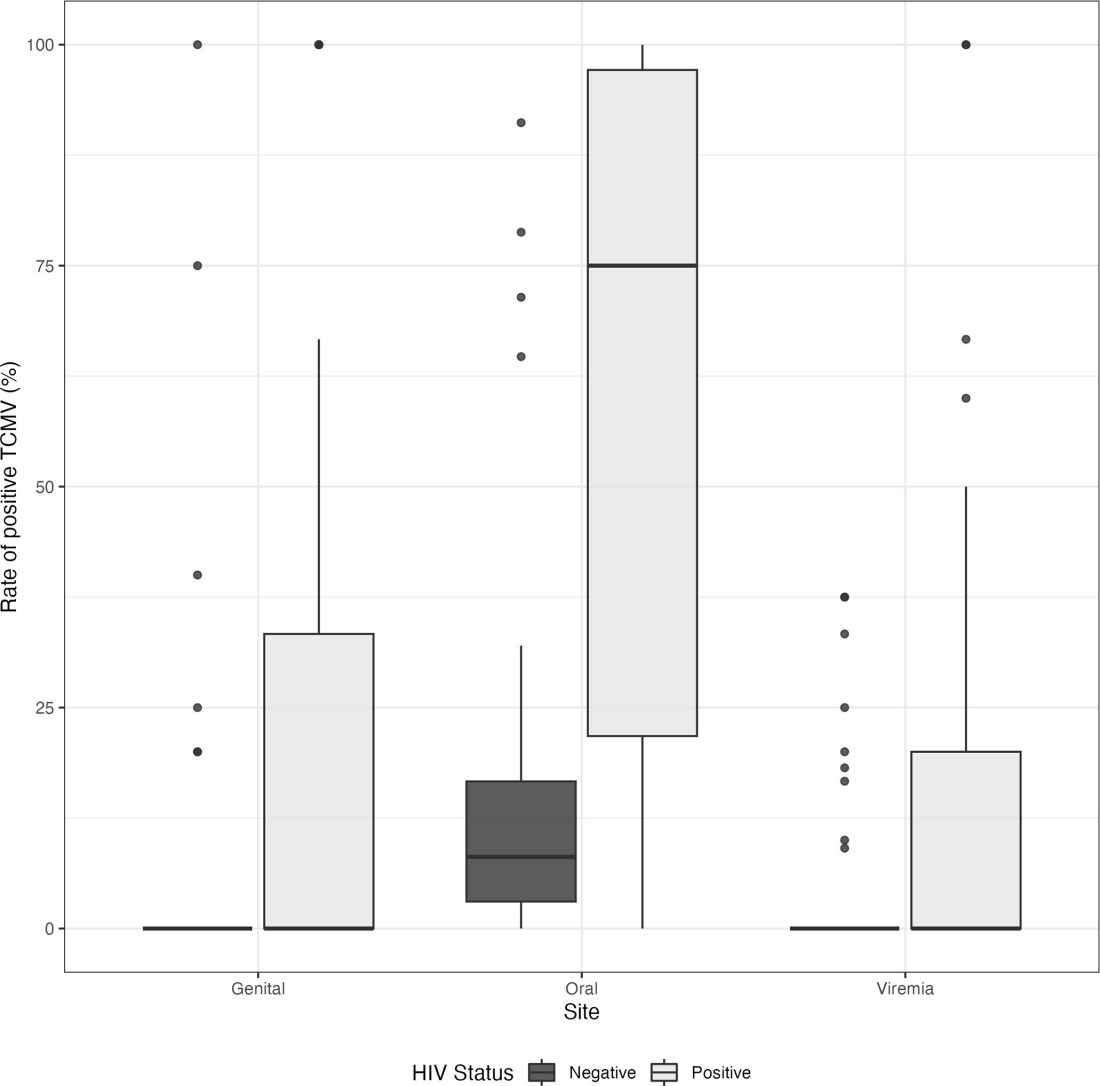
Rate of CMV shedding for all samples, by HIV status and anatomic site.

Overall, 88.4% of PLWH and 85.7% of people without HIV had oral CMV detected. Despite this, most displayed no genital shedding or viremia (Fig 1). The frequency of CMV detection was highest in oral samples compared to other anatomical sites. Among PLWH, oral CMV was detected in 61% of samples, compared to 16% of oral samples from people without HIV (Table 2). Compared to participants without HIV, PLWH had 2–4 times greater risk of CMV detection at all anatomical sites (Table 2). HIV co-infection was associated with nearly four times greater frequency of oral shedding (aRR: 3.85, 95% CI: 2.43–6.05). Among all participants, oral shedding was intermittent and rates were heterogeneous (Fig 2 and S1 Fig in S1 File). Although the absolute frequencies were lower, detection in genital swabs and plasma samples were similarly increased among PLWH (genital swabs: 20.7% vs. 5.9%, RR 3.49; plasma: 13.1% vs. 6.5%, RR 2.37; Table 2). Similarly, the mean quantity of virus detected was higher among PLWH at all anatomical sites, particularly oral swabs (Table 2).

**Table 2:** Frequency and mean quantity of site-specific CMV replication among PLWH compared to people without HIV.

**Site**	**Detection frequency**		**RR (95% CI)** *
	**HIV seropositive** **(N = 42)**	**HIV seronegative**	**-**
**Oral**	**773/1272 (61%)**	**214/1349 (16%)**	**3.85 (2.43, 6.05)**
**Genital**	**35/170 (21%)** **27/207 (13%)**	**9/154 (6%)**	**3.49 (1.39, 8.85)**
**Viremia**	**27/207 (13%)**	**15/232 (6%)**	**2.37 (1.02, 5.51)**
**Site**	**Mean CMV quantity log/mL (SD)**		Mean difference (95%) CI)**
	**HIV seropositive** **(N = 42)**	**HIV seronegative**	
**Oral**	**2.0 (1.84)**	**0.3 (0.97)**	**1.63 (1.13, 2.13)**
**Genital**	**0.5 (1.16)**	**0.1 (0.42)**	**0.42 (0.13, 0.70)**
**Viremia**	**0.3 (0.77)**	**0.1 (0.4)**	**0.21 (0.05, 0.38)**

*Estimated using a GEE log-binomial model with an assumed exchangeable correlation structure with and without adjustment for KS status.

** Estimated using a GEE model with Gaussian link with an assumed exchangeable correlation structure with and without adjustment for KS status.

Among PLWH, increased CD4+ count was not associated with decreased genital shedding or viremia (Table 3). However, each increase of 100 cells/mm^3^ was associated with an 11% decrease in oral shedding frequency. Each log increase in HIV viral load was associated with a 39% increase in CMV oral shedding frequency. There was a trend towards increased CMV viremia frequency with increasing HIV viral load, but no association with genital shedding. None of the results were modified by adjustment for KS status (S1 and S2 Tables in S1 File).

**Table 3:** Estimated effect of HIV viral load and CD4+ T-cell count on risk of site-specific CMV detection among PLWH.

	Oral shedding	Genital shedding	Viremia
CD4 count*	0.89 (0.81, 0.99)	1.00 (0.98, 1.02)	0.96 (0.92, 1.01)
Log10 HIV c/mL**	1.39 (1.07, 1.79)	0.94 (0.56, 1.60)	1.87 (0.94, 3.73)

All data are presented as risk ratios with 95% confidence intervals.

*Each 100 cells/mm^3^ increase in CD4+ T-cell count.

**Each log_10_ increase in HIV copies/mL.

## Discussion

In this prospective cohort, HIV infection was associated with more frequent and higher quantity mucosal and systemic CMV replication. Thirty-five percent of PLWH had CMV viremia, a higher proportion than in studies of AIDS patients [17,28,29], despite all participants having CD4+ T-cells >200 cells/mm^3^. Lower rates of CMV viremia in those studies may have been due to methods of detection and/or fewer samples collected per participant. Rates of CMV detection from oral swabs were also higher in the current study than some prior studies of people with or without HIV, likely due to single timepoint saliva sampling [14]. Our rate of oral CMV detection (61%) is similar to another frequently-sampled Ugandan cohort (>50%) [1,30].

The rate of CMV detection from genital swabs in PLWH was slightly lower than previously described from cervical specimens (59%) [31], vaginal specimens (35%) [19], or semen (30%) [32]. This may be because we collected a mixed anogenital swab, inclusion of participants with lower CD4+ count, or fewer samples per person.

Among PLWH in this study, HIV viral load and CD4+ count were associated with CMV oral shedding frequency, not genital replication or viremia, although there was a trend towards association between HIV viral load and CMV viremia.

A strength of this study is the intensive sampling scheme, which provides a more robust estimate of replication rates than cross-sectional or less frequent sampling [33]. Limitations include relatively small sample size and specific characteristics of the cohort. In Uganda, CMV infection is typically acquired early in life, which could affect later control of CMV replication. Furthermore, high rates of CMV viremia and shedding in this cohort might be due in part to reinfection, as transmission rates are high in Uganda [1,34,35]. These data provide insight into viral dynamics and interactions between viral co-infections in a way that could not be analyzed in a contemporary cohort in the presence of ART for all PLWH. Findings from this study can provide a high quality comparison group for future studies that take place in the context of ART or that seek to assess the impact of treatments such as letermovir.

It may not be appropriate to generalize findings from this cohort to PLWH with advanced HIV disease or those on ART. However, our findings are pertinent to the 9.6 million PLWH globally who are not accessing ART [36] and provide interesting data for further study in all populations of PLWH. Neither markers of systemic inflammation nor CMV-specific immune responses were measured, but might provide insight into mechanisms of impaired CMV control among PLWH. As CMV replication was largely independent of HIV viral load and CD4+ count, interventions beyond ART may be required to mitigate the impacts of CMV in PLWH with non-advanced HIV disease. Indeed, CMV viremia is an independent risk factor for death among PLWH receiving ART [7,8]. Rates of congenital CMV among HIV-exposed infants remain elevated despite ART and early CMV replication in infants can negatively impact the establishment of the HIV reservoir in cases where they are HIV-infected [37,38]. Thus, medications to control CMV replication, such as valganciclovir or letermovir, or prevention of CMV infection through vaccination would likely be particularly valuable for PLWH. Studies to assess both of these strategies are currently underway [39,40].

## Supporting information

S1 File Contains all supplementary tables and figures. (DOCX)

S2 File (CSV)

S3 File (CSV)
