# Supplementary material for: Increased frequency and quantity of mucosal and plasma cytomegalovirus replication among Ugandan Adults Living with HIV
Source: PLoS One. 2023 Aug 4;18(8):e0287516. doi: 10.1371/journal.pone.0287516 (PMC10403105; doi:10.1371/journal.pone.0287516)
Supplement: S1 File — (DOCX) [file pone.0287516.s001.docx]

**Table S1.** **Frequency and mean quantity of site-specific CMV replication among PLWH compared to people without HIV; KS unadjusted and adjusted models.**

| **Site** | **Detection frequency** | | **RR (95% CI)*** | **RR (95% CI), KS adjusted*** |
| --- | --- | --- | --- | --- |
|  | **HIV seropositive**  **(N=42)** | **HIV seronegative** | **-** |  |
| **Oral** | **773/1272 (61%)** | **214/1349 (16%)** | **3.85 (2.43, 6.05)** | **3.80 (2.46, 5.87)** |
| **Genital** | **35/170 (21%)**  **27/207 (13%)** | **9/154 (6%)** | **3.49 (1.39, 8.85)** | **3.47 (1.39, 8.66)** |
| **Viremia** | **27/207 (13%)** | **15/232 (6%)** | **2.37 (1.02, 5.51)** | **2.41 (1.10, 5.27)** |
|  | | | | |
| **Site** | **Mean CMV quantity log/mL (SD)** | | **Mean difference (95%) CI)**** | **RR (95% CI), KS adjusted*** |
|  | **HIV seropositive**  **(N=42)** | **HIV seronegative** |  |  |
| **Oral** | **2.0 (1.84)** | **0.3 (0.97)** | **1.63 (1.13, 2.13)** | **1.61 (1.14, 2.07)** |
| **Genital** | **0.5 (1.16)** | **0.1 (0.42)** | **0.42 (0.13, 0.70)** | **0.42 (0.13, 0.70)** |
| **Viremia** | **0.3 (0.77)** | **0.1 (0.4)** | **0.21 (0.05, 0.38)** | **0.22 (0.06, 0.38)** |

*Estimated using a GEE log-binomial model with an assumed exchangeable correlation structure with and without adjustment for KS status.

** Estimated using a GEE model with Gaussian link with an assumed exchangeable correlation structure with and without adjustment for KS status.

**Table S2.** **Estimated effect of HIV viral load and CD4+ T-cell count on risk of site-specific CMV detection among PLWH; KS unadjusted and adjusted models.**

|  | **Oral shedding** | **Genital shedding** | **Viremia** |
| --- | --- | --- | --- |
| CD4 count* | 0.89 (0.81, 0.99) | 1.00 (0.98, 1.02) | 0.96 (0.92, 1.01) |
| CD4 count, KS adjusted* | 0.91 (0.83, 1.01) | 1.00 (0.98, 1.02) | 0.97 (0.93, 1.01) |
| Log10 HIV c/mL** | 1.39 (1.07, 1.79) | 0.94 (0.56, 1.60) | 1.87 (0.94, 3.73) |
| Log10 HIV c/mL, KS adjusted** | 1.34 (1.05, 1.71) | 0.94 (0.56, 1.58) | 1.61 (0.87, 2.98) |

All data are presented as risk ratios with 95% confidence intervals.

*Each 100 cells/mm^3^ increase in CD4+ T-cell count.

**Each log_10_ increase in HIV copies/mL.

**Figure S1. Oral CMV shedding patterns for all participants.** The x axis describes the day of follow up within the study.
